# CRISPR-screen informed engineered T cell therapies

**DOI:** 10.3389/fimmu.2026.1839064

**Published:** 2026-05-29

**Authors:** Karrie Wong, Conor Calnan, Micah J. Benson

**Affiliations:** KSQ Therapeutics, Lexington, MA, United States

**Keywords:** adoptive cell therapy, CRISPR, solid tumor, T cell, TIL

## Abstract

Adoptive T cell therapies can deliver curative responses for refractory patients with B cell malignancies, yet clinical activity in solid tumors remains inconsistent. Tumor-intrinsic barriers dominating this inconsistency include the immunosuppressive solid tumor microenvironment (TME) imposing chronic inhibitory cues to T cells and the scarcity of patient-shared and uniformly expressed tumor-restricted antigens for T cells to target. CRISPR-based forward genetics screens enable mapping of the functional genome regulating T cell anti-tumor activity. Here, we review recent insights from pooled CRISPR knockout screens in T cells to define convergent targets and pathways regulating T cell anti-tumor function and align the pharmacology of engineered T cells with sequential barriers they encounter within the TME. We additionally propose a framework for CRISPR screen-enabled target prioritization and present an example of how these principles can be applied to the functional enhancement of T cells through TIL (Tumor Infiltrating Lymphocyte) therapy, which utilizes a patient’s personalized immune response against solid tumor antigens.

## 1 Functionally enhancing T cell therapies: an introduction

Engineering strategies to enhance adoptive T cell therapies fall broadly into ‘gene-on’ and ‘gene off’ approaches. Gene-on integrates activators into the T cell genome, often through viral transduction, to amplify function-enhancing signaling pathways. The discovery that chimeric antigen receptors (CARs) require a co-stimulation signaling domain for clearing CD19-expressing tumors catalyzed the search for additional enhancements including alternative signaling domains and ‘armoring’ with cytokines (1–3). These approaches can strongly enhance anti-tumor activity in pre-clinical models, with potential limitations including excessive or poorly tunable signaling of a single pathway and the uncoupling of T cell behavior from the oscillatory extrinsic signals that normally govern function.

Gene-off approaches instead remove intrinsic brakes, or checkpoints, from the extrinsic positive signals naturally governing T cell function and fate including T cell receptor (TCR), cytokine and co-stimulatory signaling. A key feature is that T cells retain the need for extrinsic signals to function, with biological cues and activation occurring in sync and in a tunable manner, although with an expanded and enhanced dynamic functional range due to checkpoint removal. This paradigm was established by the clinical successes of CTLA-4 and PD-1 checkpoint blockade (4). Despite these clinical successes, there are significant remaining unmet needs, particularly in the treatment of solid tumors, which have motivated the discovery of novel checkpoints for pharmacologically targeting as well as inactivation within cell therapies. Adoptive T cell therapies offer two inherent advantages as a therapeutic approach: the numerical benefit of infusing large numbers of tumor-specific T cells and, owing to the breadth of our existing genome editing toolkit, the ability to ‘gene off’ key signaling nodes, transcriptional regulators and epigenetic programs otherwise inaccessible to biologics or small molecules.

## 2 Adoptive T cell therapies for solid tumors

Approved adoptive T cell therapy products demonstrate both opportunity and lessons learned. CAR-T (Chimeric Antigen Receptor T cell) therapy involves engineering T cells to express a synthetic CAR recognizing a cell surface antigen on tumor cells; TCR-T (T Cell Receptor engineered T cell) therapy also involves engineering T cells but to express a TCR recognizing a tumor peptide antigen presented by HLA molecules in an MHC-restricted manner and thus targeting intracellular tumor antigens. TIL (Tumor Infiltrating Lymphocyte) therapy involves harvesting T cells which have infiltrated a patient’s tumor and possess tumor specificity, expanding ex vivo followed by reinfusion back into the patient. Currently approved products include CAR-T therapies targeting CD19 (Kymriah^®^, Yescarta^®^, Tecartus^®^, Breyanzi^®^, Aucatzyl^®^) and BCMA (Carvykti^®^, Abecma^®^) in hematological tumors, and TCR-T (Tecelra^®^) and TIL (Amtagvi^®^) therapies for synovial sarcoma and melanoma, respectively. Broader success of T cell therapies targeting solid tumors has been hampered in part by two major challenges. First, the highly immunosuppressive nature of the solid tumor microenvironment (TME) is driven by numerous mechanisms serving to blunt T cell infiltration, suppress function, and drive their differentiation towards a terminally exhausted state. The TME is characterized by hypoxic conditions, increased lactic acid and deprivation of glucose and glutamine, all of which facilitate T cell dysfunction and exhaustion (5, 6). Notably, the stringent immunosuppressive mechanisms present within solid tumors are distinct from those present with hematological malignancies, which tend to accumulate within lymphoid tissues where many of these mechanisms are not overtly apparent (7). Second, the targeting of tumors by cell therapies is hindered by a scarcity of patient-shared antigens uniquely and uniformly expressed by solid tumors and not healthy tissues, either on the cell surface or presented in the context of MHC molecules. The targeting of tumor antigens also expressed by normal tissues can lead to on-target/off-tumor toxicities, and the molecular heterogeneity of solid tumors can drive the concomitant heterogeneous expression of tumor antigens, with antigen loss a common mechanism utilized by tumors to escape immune recognition (8, 9).

The cumulative clinical and translational experience with adoptive T cell therapies has illuminated the key mechanisms critically required for their efficacy against solid tumors. First, robust post-infusion expansion and systemic accumulation of transferred cells within primary and secondary lymphoid organs should ensure that sufficient tumor-recognizing T cells are present within the circulation to initiate an acute response. Second, intratumoral T cell trafficking, infiltration and accumulation are essential for driving proximity and tumor cell contact at a sufficient effector-to-target ratio needed to kill tumor cells. Third, tumor infiltrating T cells must retain effector function and resist tumor-driven immunosuppression and exhaustion. Finally, durable persistence of T cells present in lymphoid tissues over years should provide a long-lived memory backdrop capable of clearing residual disease. Rational engineering therefore requires a comprehensive understanding of the targets and pathways governing these mechanisms.

## 3 CRISPR screens for mapping T cell anti-tumor function

Forward-genetic CRISPR screens have opened the genome to discovery, repeatedly highlighting the same core pathways as regulating T cell anti-tumor function: JAK/STAT cytokine signaling, proximal and distal TCR signaling including NF-κB and RNA stability programs, and transcriptional/epigenetic regulators of exhaustion and cell fate (**Table 1**). The convergence of these pathways across studies and their roles in regulating the various mechanisms required for T cell therapies to benefit patients provides a roadmap for engineering and a framework for prioritizing targets and pathways for the functional enhancement of T cells.

**Table 1:** Overview of T cell anti-tumor function CRISPR screens.

T cell mechanism	Reference	Library	Library size	Screen setting: *in vitro/in vivo*; human/mouse	Model	Identified genes	Identified pathways
Expansion & Accumulation	Shifrut et al., Cell (2018) (10)	Genome wide library (Brunello library)	77,441 sgRNAs/19,114 genes	*In vitro*; Human CD8^+^ T cells	CD3/CD28 stim +IL-2 with or without adenosine-mediated immunosuppression	TCEB2, SOCS1, CBLB, CD5, CDKN1B, DGKA, DGKZ, RASA2, TNFAIP3 (A20), UBASH3A	TCR signaling, Cell Cycle, Cytokine/JAK-STAT, NF-κB
	Sutra Del Galy et al., Sci Immunol (2021) (11)	Custom genome-wide library	~90,000 sgRNAs/18,400 genes	*In vivo*; Antigen-experienced murine Marilyn TCR-Tg CD4^+^ T cells	T cell adoptive transfer with *in vivo* priming	SOCS1	Cytokine/JAK-STAT, TCR signaling
	Schlabach et al., J Clin Invest (2023)* (12)	Custom library	~56,408 sgRNAs/5,137 genes	*In vitro*; Primary human Melanoma TIL	Standard Rapid Expansion Protocol (OKT3+iPBMC+IL-2)	SOCS1	Cytokine/JAK-STAT, TCR signaling
	Knudson et al., Nature (2025)* (13)	Focused 135 gene library	~1,180 sgRNAs/135 genes	*In vitro* and *in vivo*; Human BCMA-CAR-T	CD3/CD28 stim +IL-2 or IL-7/IL-15; RPMI-8226	CDKN1B, ETS1, PTPN2, RC3H1 (Roquin-1), SOCS1, TNFAIP3 (A20), ZC3H12A (Regnase-1)	Cell Cycle, Exhaustion/Transcriptional control, TCR signaling, Cytokine/JAK-STAT, NF-κB, mTORC1
	Zhao et al., Cell Reports (2021) (14)	Custom genome-wide library	78,633 sgRNAs/19,674 genes	*In vitro* and *in vivo*; Murine OT-1 CD8^+^ T cells	OVA activated OT1 expansion *in vitro* in IL-2 or *in vivo*	RC3H1 (Roquin-1), PTPN2, SOCS1, ZC3H12A (Regnase-1)	TCR signaling, Cytokine/JAK-STAT, Exhaustion/Transcriptional control, NF-κB, mTORC1
Infiltration & Intratumoral Accumulation	Datlinger et al., Nature (2025) (15)	Genome-wide library (Brunello library); focused 39-gene library	77,441 sgRNAs/19,114 genes (*in vitro*); 339 sgRNAs/39 genes (*in vivo*)	*In vitro* and *in vivo*; Human CD19-CAR-T	CD3/CD28 or CD19+ K562 stim; Nalm6	CTLA4, FAS, PRDM1 (Blimp-1), RHOG, RHOG+FAS (combination)	Immune checkpoint, Exhaustion/Transcriptional control, Migration/cytoskeleton
	Liu et al., bioRxiv (2025) (16)	Genome-wide library (Brunello library)	~77,441 sgRNAs/~19,114 genes	*In vivo*; Primary human T cells	Membrane-bound anti-CD3-expressing A375	MAP4K1 (HPK1), P2RY8, PTGER4, SOCS1, TNFAIP3, (A20), ZC3H12A (Regnase-1)	TCR signaling, MAPK, Migration/cytoskeleton, Cytokine/JAK-STAT, NF-κB, Exhaustion/Transcriptional control, mTORC1
	Ye et al., Nat Biotechnology (2019) (17)	Focused cell surface receptor library	~7,628 sgRNAs/~1,657 genes	*In vivo*; Murine CD8^+^ T cells	GL261	PDIA3, MGAT5, EMP1, LAG3, MGAT5+PDIA3 (combination)	Migration/cytoskeleton, Immune checkpoint, TCR signaling, Antigen presentation
	Wei et al., Nature (2019) (18)	Focused metabolic library; genome-wide library (Brie library)	Metabolic: 9,551 sgRNAs/~3,017 genes; Brie: 78,637 sgRNAs/~19,674 genes	*In vivo*; Murine OT-1 CD8^+^ T cells & Regnase-1 null OT-1 CD8^+^ T cells	B16-OVA	PTPN2, SOCS1, ZC3H12A (Regnase-1), ZC3H12A+PTPN2, ZC3H12A+SOCS1	TCR signaling, Cytokine/JAK-STAT, NF-κB, Exhaustion/Transcriptional control, mTORC1
	Schlabach et al., J Clin Invest (2023)* (12)	Focused library of T cell related genes	~14,237 sgRNAs/1,373 genes	*In vivo*; Murine OT-1 or PMEL CD8^+^ T cells	B16-OVA and MC38-gp100	SOCS1, CBLB, NFKBIA (IκBα), NR4A1, NR4A2, NR4A3, PDCD1 (PD-1), PTPN2, TGFBR1, TGFBR2	Cytokine/JAK-STAT, TCR signaling, NF-κB, Exhaustion/Transcriptional control, Immune checkpoint, TGF-β signaling
	Milling et al., J Exp Med (2024) (19)	Focused 100-gene FITS library (UMI-barcoded sgRNAs)	~100 genes (multi-sgRNA, UMI-incorporated for clonal tracking)	*In vivo*; Murine OT-1 CD8^+^ T cells	B16-OVA	CBLB, FLI1, PDCD1 (PD-1), PTPN1, PTPN2, ZC3H12A (Regnase-1)	TCR signaling, Exhaustion/Transcriptional control, Immune checkpoint, Cytokine/JAK-STAT, NF-κB, mTORC1
	Lafleur et al., Nat Immunol (2025) (20)	Custom druggable-genome library (focused)	2,747 sgRNAs/899 genes	*In vivo*; Murine OT-1 CD8^+^ T cells	B16-OVA	CHIC2, DGKZ, ETS1, PDCD1 (PD-1), PTPN1, PTPN2, STUB1, TGFBR1, TGFBR2, TNFAIP3 (A20)	TCR signaling, NF-κB, Exhaustion/Transcriptional control, Immune checkpoint, Cytokine/JAK-STAT, TGF-β signaling
	Mercier et al., bioRxiv (2026)* (21)	Custom genome-wide library + focused hitlist pairwise combinations library (CRISPR^2)	~170,000+ sgRNAs/21,422 genes; focused combinatorial hitlist 74 sgRNAs/36 genes	*In vivo*; Murine OT-1 and PMEL CD8^+^ T cells	B16-OVA, EG7-OVA, and MC38-gp100	CBLB, CHIC2, DGKZ, GNAS, IKZF1 (IKAROS), NFKBIA (IκBα), NR4A3, PDCD1 (PD-1), PELI1 (Pellino-1), PTPN1, PTPN2, RC3H1 (Roquin-1), SOCS1, STUB1, TGFBR1, TGFBR2, TNFAIP3 (A20), ZC3H12A (Regnase-1), SOCS1+NFKBIA, SOCS1+RC3H1, SOCS1+ZC3H12A (combinations)	TCR signaling, NF-κB, Exhaustion/Transcriptional control, MAPK, Cytokine/JAK-STAT, mTORC1, TGF-β signaling
Immuno-suppression	Carnevale et al., Nature (2022) (22)	Genome-wide (Brunello library)	77,441 sgRNAs/19,114 genes	*In vitro*; Primary human CD8^+^ T cells	CD3/CD28 stim +/- TGF-β, adenosine, PGE2	CBLB, CDKN1B, DGKA, RASA2, SOCS1, TGFBR1, TGFBR2, TNFAIP3 (A20)	TCR signaling, Cell Cycle, Cytokine/JAK-STAT, TGF-β signaling, NF-κB
Cytotoxic Effector Function	Wang et al., Cancer Discovery (2021) (23)	Genome-wide (Brunello library)	77,441 sgRNAs/19,114 genes	*In vitro*; Human IL13Rα2 CAR-T	Patient-derived glioblastoma stem cells co-culture	EIF5A, IKZF2 (HELIOS), TLE4, TMEM184B	Translation/Protein synthesis, Exhaustion/Transcriptional control, Endolysosomal
	Liu et al., bioRxiv (2025) (16)	Genome-wide (Brunello library)	77,441 sgRNAs/19,114 genes	*In vivo*; Primary human T cells	Membrane-bound anti-CD3-expressing A375	CBLB, DGKZ, GNAS, MAP4K1 (HPK1), RASA2, SOCS1, STUB1, TNFAIP3 (A20), ZC3H12A (Regnase-1)	TCR signaling, MAPK, Cytokine/JAK-STAT, NF-κB, Exhaustion/Transcriptional control, mTORC1
	Dong et al., Cell (2019) (24)	Genome-wide library	129,209 sgRNAs/~20,611 genes	*In vivo* & *in vitro*; Murine OT-1 CD8^+^ T cells	E0771-OVA & co-culture	DHX37	NF-κB
	Chen et al., Cell (2021) (25)	Focused transcription factor library	~480 sgRNAs/~120 Transcription Factor Genes	*In vivo*; Murine P14 CD8^+^ T cells	Chronic LCMV (Clone13) infection	FLI1	Exhaustion/Transcriptional control
Exhaustion	Freitas et al., Science (2022) (26)	Genome-wide library (Bassik library)	~211,695 sgRNAs/~20,500 genes	*In vitro*; Human GD2 CAR-T with and without HA.28z (tonic signaling)	Nalm6-ALL-GD2-GFP co-culture	MED12, CCNC (Cyclin C)	Exhaustion/Transcriptional control, Cell Cycle, Apoptosis
	Belk et al., Cancer Cell (2022) (27)	Genome-wide library; focused mini-pool libraries	~90,230 sgRNAs/~20,000 genes (primary); focused 2,000 sgRNAs/300 gene mini-pool for *in vivo*	*In vitro*; Murine CD8^+^ T cells; *in vivo* OT-1 CD8^+^ T cells	Chronic re-stim with CD3 & IL2 or IL2 alone; MC38-OVA, B16-OVA	ARID1A	Epigenetics/Chromatin
	Tay et al., Cell Reports Medicine (2024) (28)	Focused epigenetic library	~2,487 sgRNAs/829 epigenetic-regulator genes	*In vitro*; Primary human T cells	Chronic CD3/CD28 stimulation	IKZF1(IKAROS)	Exhaustion/Transcriptional control
	Fumagalli et al., Molecular Therapy (2026)* (29)	Focused hitlist library	236 sgRNAs/50 genes	*In vitro*; Human EGFR-CAR-T cells (with 41BB or CD28 co-stim domain)	Chronic CD3/CD28 or (EGFR antigen stim	SOCS1, ZC3H21A (Regnase-1), PTPN2, RASA2, DGKA, DGKZ, CD5	TCR signaling, Cytokine/JAK-STAT, NF-κB, Exhaustion/Transcriptional control, mTORC1
	Zhou et al., Nature (2023) (30)	Focused transcription factor library	~800 sgRNAs/~180 transcription factors	*In vivo*; Murine OT-1 CD8^+^ T cells	B16-OVA	ETS1, IKZF1 (IKAROS), RBPJ	Exhaustion/Transcriptional control
	Trefny et al., Nat Commun (2023) (31)	Focused 32 exhaustion-DEG genes library	~180 sgRNAs/32 genes	*In vitro*; Human NY-ESO-1 TCR-T	Chronic re-stim with T2 antigen presenting cells pulsed with NY-ESO peptide	PTPN2, SNX9, TGFBR2	TCR signaling, Cytokine/JAK-STAT, Migration/cytoskeleton, TGF-β signaling, Exhaustion/Transcriptional control
Persistence & Memory	Wang et al., J Exp Med (2024) (32)	Genome-wide library (Brie library in Regnase-1 KO background)	~78,000 sgRNAs/~19,674 genes (in Regnase-1 KO context)	*In vivo*; Murine CD19-CAR-T cells	Adoptive transfer into C57B/6 host	BCOR + ZC3H12A (Regnase-1) (combination)	Epigenetics/Chromatin, Exhaustion/Transcriptional control, NF-κB, TCR signaling, mTORC1

*Indicates publications that included long-duration (>30 days) *in vivo* screening or validation experiments. These extended time frames provide experimental context relevant to assessing T cell persistence and memory. stim, stimulation; re-stim, re-stimulation.

Pooled CRISPR knockout screens introduce libraries of single guide RNAs (sgRNAs) into tumor-specific T cells such that each cell harbors a distinct perturbation identifiable by its integrated sgRNA (33, 34). Gene-edited T cells are then functionally evaluated. Enrichment of specific sgRNAs reveals genes whose loss enhances the assayed function and depletion marks positive regulators or genes essential for cell survival. A feature of this approach is its flexibility. Screens can be conducted both *in vitro* and *in vivo*, and have been used to map proliferation and accumulation, cytokine production and degranulation as proxies for cytotoxicity, resistance to tumor-derived immunosuppression, survival and apoptosis, exhaustion and *in vivo* tumor infiltration as well as persistence (**Table 1**). Recovery of sgRNAs from assayed T cells at a depth predefined by statistical power drives screen quality and is a primary determinant of sgRNA library size.

*In vitro* screens excel at variable control by precisely isolating mechanisms under inquiry. In addition, they allow scale-up screens for genome-wide coverage, given the ability to recover tens of millions of assayed T cells. Assayed mechanisms include T cell expansion and accumulation, including under immunosuppressive conditions, effector function, and functional exhaustion. The primary limitation of *in vitro* screens is ecological. That is, *in vitro* settings cannot fully capture the emergent properties of an intact TME, including T cell trafficking and intratumoral accumulation, stromal and myeloid cell crosstalk, and the spatial nutrient and oxygen gradients present. It is plausible that important targets may be missed. Indeed, in all *in vitro* T cell CRISPR screens conducted to date, the canonical checkpoint PD-1 has not emerged as a hit, underscoring ecological gaps (**Table 1**). Ultimately, *in vitro* hits should be validated *in vivo*, with the value of *in vitro* CRISPR screens intimately coupled to *in vivo* validation.

*In vivo* screens evaluate T cell function within the physiological context of tumor-bearing hosts, with intratumoral T cell accumulation commonly used as the primary mechanism in solid tumor models. Though not a direct measure of anti-tumor activity, T cell intratumoral accumulation encompasses several mechanisms required for fulminant T cell anti-tumor function and has demonstrated its utility as an appropriate proxy across studies, as discussed below. These settings effectively model tumor-driven selective pressure on T cells and have revealed novel checkpoints and pathways that constrain effective immunity. The main limitation of *in vivo* solid tumor screens is that of low T cell recovery from tumors. Depending on the model screened, it is typical to obtain 1,000-10,000 transferred T cell clones per tumor rather than the tens of millions often obtainable *in vitro*. For this reason, many *in vivo* T cell screens have relied on the use of focused sgRNA libraries targeting a defined gene class with hundreds or a few thousand genes in total. This has motivated staged approaches wherein genome-scale *in vitro* screens are followed by targeted *in vivo* screens (27), as well as methods to improve resolution and to scale-out screening scope (27). Distinct from accumulation screens, single-cell CRISPR ‘Perturb-Seq’ screens couple perturbations to transcriptional state, adding resolution on fate trajectories, albeit with lower throughput and higher analytical complexity (35, 36).

Targets uncovered *in vivo* depend on T cell context, tumor model and the host mouse strain. Syngeneic as well as human xenograft tumor models have been utilized (**Table 1**), with each setting providing insights relevant to the specific aspect of T cell biology being interrogated. For syngeneic models, the transfer of TCR-transgenic (TCR-Tg) T cells, such as OT1s, have been widely used, with their main benefit the ability to conduct screens in mice within a context of immunocompetency to model the cellular complexities of the TME. In contrast, human xenograft models are typically conducted in immunodeficient mice yet afford the benefit of modeling human CAR-T and TCR-T cell biology within human tumor contextures suited for elucidating intrinsic constraints in T cells in relation to xenograft type and the specific CAR/TCR of choice. It is of note that particular tumor types may rely more or less on a particular suite of immunosuppressive mechanisms, impacting the nature of inactivated genes and pathways enhancing anti-tumor function. In addition, CAR design features, including CAR affinity/avidity, clustering, thermodynamic stability, hinge and co-stimulation all may influence the nature of genes identified. CRISPR screen breadth across species, T cell therapy types and models provide complementary mechanistic views that together chart a practical route to engineering. Below, we highlight key CRISPR screen-informed targets and pathways.

## 4 CRISPR-screen informed genes and pathways facilitating the enhancement of T cell therapies

**Figure 1:**
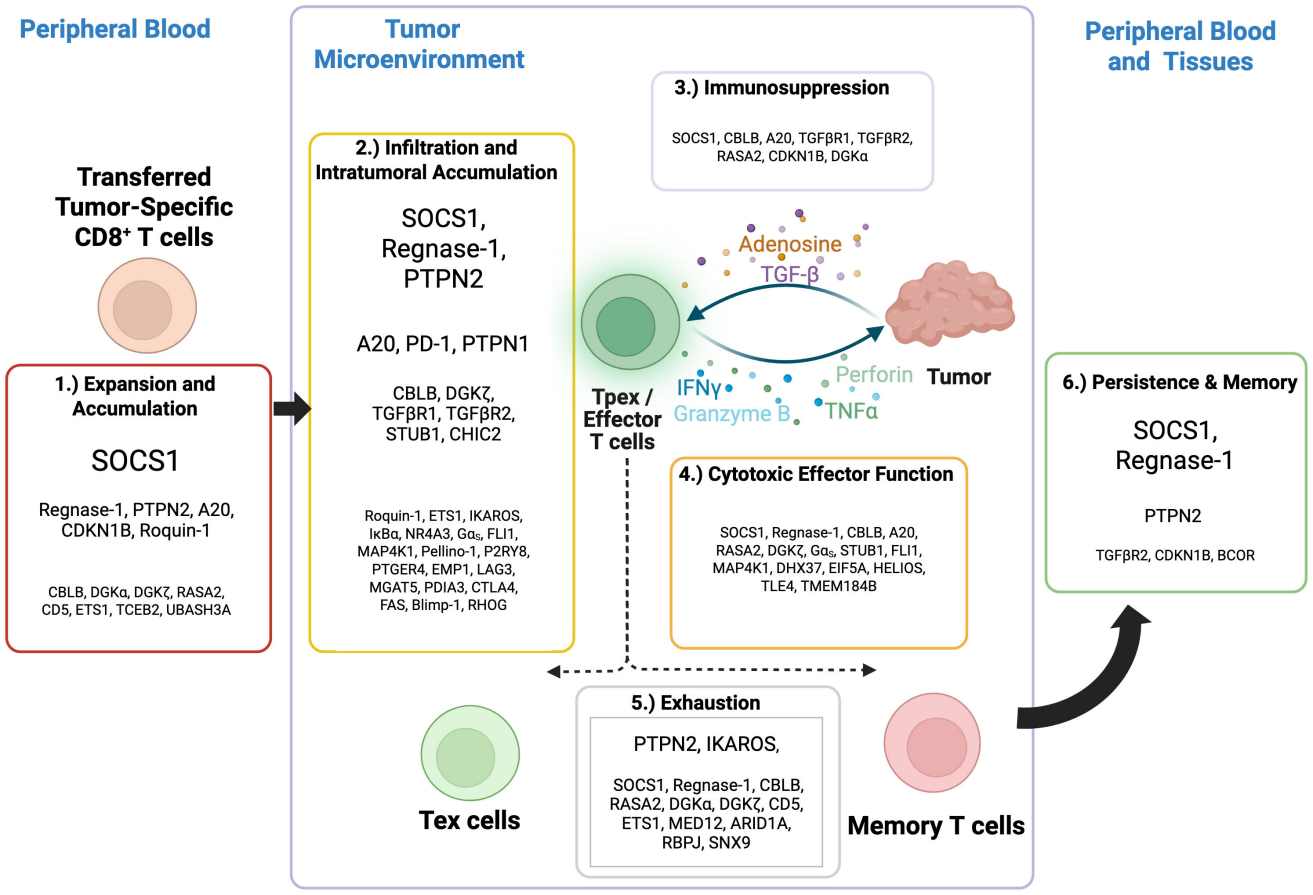
Overview of validated hits identified in CRISPR screens assessing T cell function as categorized by mechanism. Font size indicates the number of independent CRISPR screen publications that a hit was identified (regular font: n=1 screen; medium font: n=2–3 screens; large font: n=4 screens; jumbo font: 5 screens (see **Table 1**). Hits reported in CRISPR screens (**Table 1**) found to enhance adoptive T cell anti-tumor function in mouse *in vivo* tumor studies independent of the reported screen study were included for completeness and include A20 (37), Blimp-1 (38), CBLB (39), CD5 (40), CTLA-4 (41), DGKα (42), DGKζ (42), NR4A1-3 (43), MAP4K1 (44), Pellino-1 (45), PTGER2 (46), PTPN1 (47), TGFβR1-2 (48), and UBASH3A (49). Created in BioRender. Benson, M. (2026) https://BioRender.com/ntv14jr.

A compilation of CRISPR screens aimed at enhancing T cell anti-tumor function identifies the most frequently recovered and validated targets and pathways (**Table 1**). The mechanisms span i) expansion and systemic accumulation; ii) tumor infiltration and intratumoral accumulation; iii) resistance to tumor immunosuppression; iv) cytotoxic effector function; v) exhaustion and differentiation fate and vi) persistence and memory formation (**Figure 1**). Below, we summarize key genes and pathways regulating each of these mechanisms and suggest how they can inform engineering strategies.

### 4.1 Expansion and systemic accumulation

Early expansion in blood is a consistent correlate of response in hematologic CAR-T therapies and a prerequisite for activity in solid tumors (50, 51). Across genome scale and focused CRISPR screens in both human and murine systems, negative regulators of cytokine and TCR signaling repeatedly emerge as key constraints of this phase. The first genome-wide *in vitro* proliferation screens in human T cells identified inhibitors of proximal TCR and NF-κB signaling, including RASA2, CBLB, CD5, DGKζ, UBASH3A and A20 (encoded by *TNFAIP3*), along with cytokine pathway brakes such as SOCS1 and its adaptor TCEB2 (10). The Ras GTPase RASA2 dampens TCR signaling by accelerating RAS-GTP hydrolysis and attenuating RAS pathway output (10). Independent studies found that RASA2 and SOCS1 limit *in vitro* accumulation of BCMA CAR-T cells (13); SOCS1 similarly constrained TIL expansion under manufacturing conditions (12). Genome-wide screens in murine CD8^+^ OT1 TCR-transgenic T cells identified Roquin-1 (encoded by *RC3H1*), Regnase-1 (encoded by *ZC3H12A*) and PTPN2 as strong negative regulators of *in vitro* proliferation; Roquin-1, Regnase-1 and SOCS1 likewise constrained *in vivo* splenic accumulation following transfer (14). SOCS1 also emerged as the top hit in *in vivo* genome-wide accumulation screens using murine CD4^+^ TCR-transgenic T cells (11). *In vivo* CRISPR screens using BCMA CAR-Ts targeting disseminated hematologic malignancies identified PTPN2, Regnase-1, and Roquin-1 as early constraints on post-transfer CAR-T expansion, while p27Kip1 (encoded by *CDKN1B*), PTPN2, SOCS1, SMARCB1 and Regnase-1 limited accumulation at later timepoints (13).

Collectively, and despite differences in species, screening modalities and disease models, negative regulators of cytokine and TCR signaling including SOCS1, PTPN2, Regnase-1 and Roquin-1 are repeatedly identified as brakes on T cell accumulation (**Figure 1**). SOCS1 suppresses signaling downstream of cytokines including IL-2, IL-7, IL-12, IL-15 and IL-21 through dampening of JAK1/2 signals, while PTPN2 negatively regulates JAK1/3 as well as TCR signaling (52, 53). Regnase-1 and Roquin-1 are critical nodes regulating mRNA abundance of regulators involved across multiple inflammatory pathways including ICOS and IL-6 and through direct inhibition of canonical NF-kB signaling through targeting of c-Rel (54–56).

### 4.2 Tumor infiltration and intratumoral accumulation

Effective infiltration and accumulation of T cells within solid tumors is critical for efficacy and a strong correlate of response to immunotherapies (57–59). While tumor accumulation reflects many of the same mechanisms driving systemic expansion, there are additional processes involved including tumor trafficking and infiltration, antigen-driven proliferation and survival while resisting immunosuppression and terminal exhaustion. As *in vitro* systems cannot as yet faithfully model tumor infiltration and spatial tumor ecology, intratumoral accumulation screens have been performed almost exclusively *in vivo*. Limited recovery of T cells from tumors imposes constraints on library size and screening power, with studies relying primarily on the use of focused libraries. Substantial method development is required to perform intratumoral T cell accumulation screens at high resolution, as detailed by FITS, a published framework for *in vivo* T cell screens (19). Genome-scale insights can be achieved through specialized methods. These include subdivision of genome-wide libraries into smaller “bookshelves” followed by aggregation of bookshelf data to obtain genome-wide insights or by using experimental strategies to enhance the accumulation of T cells within tumor such as performing screens against a backdrop of a tumor-accumulating single edit or by engineering tumor lines to retain infiltrating T cells (16, 18, 21, 32).

Foundational work in the OT1/B16-OVA model using a focused shRNA (short hairpin RNA) library identified TCR pathway inhibitors such as CBLB and DGKζ, as well as the PP2A phosphatase family PPP2R2D (60). CRISPR/Cas9 screening has since demonstrated improved perturbation fidelity over shRNA-based approaches (61). *In vivo* CRISPR screens in the OT1/B16-OVA model subsequently identified Regnase-1 as a strong negative regulator of CD8^+^ T cell tumor accumulation, with dual-inactivation of Regnase-1 with SOCS1 or PTPN2 producing strong combination effects (18). Mechanistically, Regnase-1 restrained T cell accumulation in part through targeted degradation of BATF transcripts (18).

Additional *in vivo* CRISPR screens in chronic infection and tumor models further implicated PTPN2 as a regulator of both tumor accumulation and exhaustion (62, 63). Orthotopic tumor models identified checkpoint molecules (PD-1, TIM-3) and the novel regulator PDIA3 as constraints (17, 24). Methodological advances such as the use of Unique Molecular Identifiers (UMIs) and the subdivision of libraries into “bookshelves” have enabled improved *in vivo* screen resolution, including genome-wide insights (19, 21). Screens conducted using multiple types of TCR transgenic T cells in syngeneic models consistently surface SOCS1, Regnase-1, IκBα, PTPN2 and Roquin-1 as dominant intrinsic brakes on intratumoral accumulation (12, 19, 21). Additional *in vivo* screens using OT1s as well as human T cells further converged on JAK/STAT signaling via the E3 ubiquitin ligase STUB1-mediated inhibition of IL-27α receptor expression levels (16, 20, 21). Confirming and extending the identification of SOCS1 and PTPN2 as top Regnase-1 combination partners, additional combinatorial screens ranked SOCS1/Regnase-1, SOCS1/Roquin-1 and SOCS1/IκBα as top dual edits, with SOCS1/Regnase-1 yielding the strongest activity (18, 21). Cross-species concordance was observed in an *in vivo* EGFR CAR-T/A549 lung model CRISPR screen that again recovered Regnase-1, SOCS1, and PTPN2 (29). A genome-wide human T cell *in vivo* screen against melanoma cells engineered with membrane-associated anti-CD3 enriched for Regnase-1, SOCS1 and A20, and additionally identified P2RY8 as an inhibitor of intratumoral T cell trafficking, implicating GPCR signaling (16). Complementary two-step *in vitro*/*in vivo* screen workflows identified the GTPase RHOG and the death receptor FAS as constraining CD19 CAR-Ts *in vivo* accumulation, with dual-inactivation enhancing proliferation, central memory differentiation, and efficacy (15).

**Figure 2:**
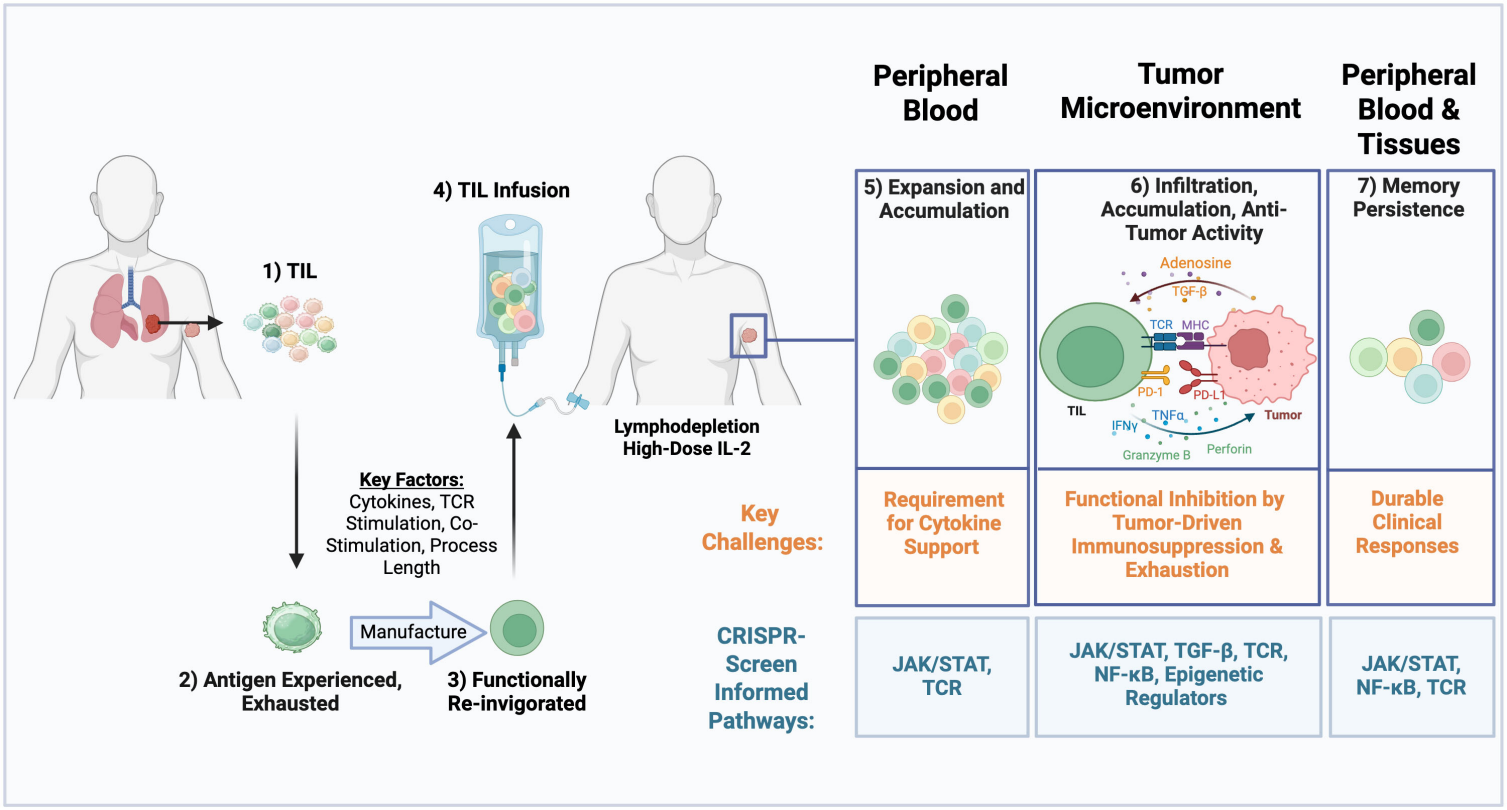
Applying insights from CRISPR screens to overcome TIL therapy challenges. Tissue resident polyclonal tumor reactive TIL (TRT) can be harnessed through an ex vivo expansion process as a personalized cell therapy for solid tumor treatments. TIL are chronically exhausted, and despite functional re-invigoration through the manufacture process, face a multitude of challenges once infused *in vivo*, including (1) expansion/accumulation to meaningful numbers in peripheral blood, which requires cytokine support from lymphodepletion and high-dose IL-2 infusion; (2) infiltration, expansion/accumulation and mediation of anti-tumor effector activities in the immunosuppressive tumor microenvironment; and (3) differentiation into memory subsets with long-term persistence. Insights from CRISPR screens inform potential target pathways to enable TIL to overcome each of these challenges. Created in BioRender. Benson, M. (2026) https://BioRender.com/ku2bn7u.

Across studies, the strongest and most reproducible regulators of intratumoral T cell accumulation include SOCS1, PTPN2, Regnase-1 and Roquin-1, which are core negative regulators of cytokine and TCR signaling (**Figure 2**). While there is target overlap between systemic accumulation screens conducted in hematological models and solid tumor accumulation models, intratumoral accumulation as a mechanism is particularly relevant towards enhancing T cell function against solid tumors. Many of the identified genes regulating tumor accumulation additionally intersect with pathways governing immunosuppression, exhaustion, and persistence as discussed below, underscoring their central role in limiting effective T cell accumulation within tumors.

### 4.3 Resistance to tumor immunosuppression

Tumors suppress T cell function through multiple mechanisms, including immunosuppressive cytokines (e.g., TGF-β), hypoxia, induction of Foxp3^+^ regulatory T cells, and metabolite accumulation (64). Genome-wide CRISPR screens identified several intrinsic checkpoints whose disruption confers resistance, including SOCS1, CBLB and A20 as well as the Ras GTPase RASA2 (22). RASA2 was also identified as a negative regulator of *in vitro* T cell accumulation, with inactivation increasing antigen sensitivity, effector function and anti-tumor activity of CAR-T and TCR-T cells across hematological and solid tumor models (22). However, benefits can be context-dependent, as no efficacy enhancement was observed upon RASA2 deletion in a disseminated *in vivo* multiple myeloma model, despite enhancing *in vitro* expansion (13). Collectively, these findings establish RASA2, along with SOCS1, CBLB and A20 as key regulators of tumor-driven immunosuppression and highlight the central role of cytokine and TCR signaling in shaping T cell anti-tumor efficacy.

### 4.4 Cytotoxic effector function

The capacity of T cells to directly lyse tumor cells constitutes the functional endpoint of effective anti-tumor immunity. Effector function can be proxied through use of surrogate biomarkers including production of IFNγ, TNFα and granzymes as well as CD107a surface mobilization as a measure of degranulation. Genome-wide *in vitro* CRISPR screens assaying for CD107a identified the DEAH box RNA helicase DHX37 as a negative regulator of degranulation, its loss enhanced the anti-tumor activity of murine CD8^+^ T cells (24). In IL-13Rα2 CAR-T cell CRISPR screens targeting glioblastoma stem cells, low PD-1 expression as an effector proxy identified TLE4 and HELIOS (encoded by *IKZF2*) as suppressors of cytotoxicity (23). *In vivo* genome-wide screens using IFNγ production as a biomarker to assay for functional tumor-infiltrating T cells confirmed RASA2, DGKζ, CBLB and STUB1 as intrinsic constraints and additionally identified Gα_s_ (encoded by *GNAS*) as an additional negative regulator (16). Gα_s_ is the stimulatory alpha subunit of a heterotrimeric G protein complex mediating GPCR signaling and promotes CD8^+^ T cell dysfunction and additionally scored in OT1/B16-OVA screens (21, 65). Chronic infection CRISPR screens identified FLI1 as a repressor of effector differentiation, with validation in tumor models demonstrating enhanced accumulation of FLI1-deficient tumor-specific CD8^+^ T cells (66). FLI1 inactivation was additionally observed to enrich in OT1/B16-OVA screens (19, 21). Collectively, these studies underscore how regulators of effector function frequently converge with pathways governing T cell accumulation and anti-tumor efficacy.

### 4.5 Exhaustion and differentiation fate decisions

Sustained antigen exposure in the TME drives T cells into a terminally differentiated exhausted (Tex) state characterized by blunted anti-tumor activity; preventing or reversing this trajectory is a central goal for immunotherapies. *In vitro* exhaustion models are driven by chronic stimulation of T cells through the TCR by repeated anti-CD3 or serial tumor cell addition, or via CAR designs that trigger tonic signaling (67, 68). These systems have enabled genome-wide discovery. A GD2 CAR possessing tonic signaling and driving transcriptional and epigenetic features of exhaustion facilitated the identification of Mediator-complex regulators (MED12, CCNC) as limiting T cell expansion (26, 69, 70). MED12 loss increased cytokine signals in T cells by boosting STAT5/STAT1 activity and IL2RA expression, leading to enhanced efficacy across multiple CAR-T and TCR-T leukemia and solid tumor models (26). Benefits were, however, context-dependent, as no enhancement in anti-tumor activity was observed in an EGFR CAR-T lung tumor model (29). Additional chronic-stimulation screens have implicated SNX9, the chromatin remodeling BRG1/BRM-associated factors (BAF) complex (*ARID1A*, *SMARCD2*, and *SMARCC1*), the INO80 complex (*ACTR5*) and the transcriptional and epigenetic regulator IKAROS (*IKZF1*) (27, 28, 31). Collectively, chronic-stimulation CRISPR screens faithfully recapitulate the functional and epigenetic features of exhaustion and have served as an effective *in vitro* screening system to discover a diverse set of targets regulating T cell exhaustion.

T cell exhaustion unfolds along a differentiation continuum from precursor-exhausted (Tpex) to terminal Tex states (71, 72). Tpex cells express the transcription factor TCF1 (encoded by *TCF7*), which drives stemness and the ability to respond to tumor (25, 73–75). The presence of Tpex cells correlates with clinical responsiveness to clinical PD-1 blockade as inhibition drives their differentiation and accumulation into effector-function sufficient subsets (76). Loss of TCF1 expression and increased expression of TOX promotes differentiation of Tpex towards Tex subsets that have varying proliferative and effector capabilities; terminally differentiated and functionally exhausted cells is the ultimate fate of the Tex lineage (77–82). Single cell Perturb-Seq CRISPR screens have mapped regulators of these transitions, adding a new dimension of insight into Tex fate decisions (35, 36). For example, while IKAROS was identified as a brake on *in vitro* T cell exhaustion and intratumoral accumulation in pooled screens, Perturb-Seq readouts provided the added dimension of demonstrating that IKAROS loss traps cells in a Tpex state (21, 28, 30). In this same study, the transcriptional regulator ETS1 restricted Tpex to Tex differentiation, and RBPJ restrained Tex progression towards terminal Tex (30). In chronic infection, Perturb-Seq screens uncovered KLF2 as limiting TOX-driven terminal exhaustion of Tex cells to support effector differentiation (83). While Perturb-Seq CRISPR screens have been limited in scope to date, scaling will undoubtedly lead to additional insights into T cell fate decisions.

Additional genes identified in intratumoral T cell accumulation screens that impact Tpex to Tex differentiation include Regnase-1, whose inactivation elevates TCF1 expression and expands Tpex cells due to its direct targeting of *TCF7* transcripts for degradation (84). Expansion of Regnase-1 inactivated Tpex cells similarly increases the accumulation of effector-competent Tex cells which exhibit lower TOX expression, and with the progression of Tex cells into a terminally exhausted state inhibited (18, 21, 84). SOCS1-dependent cytokine signals have additionally been identified as key checkpoints of Tpex to Tex fate, with SOCS1 inactivation as well as augmented IL-2/STAT5 signaling facilitating Tpex differentiation into effector function-sufficient Tex cells (12, 85–87). SOCS1 loss additionally increases T central memory cell accumulation (12).

With respect to design considerations of enhanced T cell therapies, candidate edited genes should confer resistance to chronic stimulation and epigenetic exhaustion. They should also favorably reshape the Tpex/Tex landscape *in vivo* through expanding Tpex and effector-capable Tex while limiting terminal exhaustion. Detection of Tpex/Tex subsets by phenotype has been established in murine T cell models yet less so in human CAR-T/TCR-T models: for the latter, the ultimate test is one of functionality, ensuring that enhancements facilitate stemness and effector-function while mitigating exhaustion.

### 4.6 Persistence and memory formation

Durable benefit from adoptive T cell therapies requires long-term persistence. CRISPR screens of T cell persistence *in vivo* are challenging due to the considerable sgRNA library bottlenecking that occurs following the accumulation peak and then contraction of responding T cell populations, with persistence typically assessed weeks to months after transfer. Nevertheless, *in vivo* CRISPR screens have successfully identified intrinsic checkpoints of persistence. In BCMA CAR-T models, inactivation of PTPN2 and CDKN1B enhanced efficacy, accumulation and persistence for up to 80 days following transfer (13). Similarly, in an EGFR CAR-T solid tumor model, Regnase-1, PTPN2 and SOCS1 inactivation increased intratumoral CAR-T accumulation out to 35 days (88). As pathways regulating acute T cell expansion and tumor killing may differ from those sustaining long-term persistence and memory, combination CRISPR screens have layered edits on backgrounds that enhance early accumulation, such as Regnase-1 loss. In a murine CD19-CAR-T model, Regnase-1 deletion drove robust early expansion followed by pronounced contraction, creating a sensitized context for identifying genes that enhanced persistence (32). These efforts identified BCOR as a dominant persistence-enhancing partner, with dual-editing of Regnase-1/BCOR enabling long-term maintenance of Regnase-1-edited CD19 CAR-T cells across tumor and chronic LCMV models through driving an ‘immortal’ cytotoxic TCF1^+^ Tpex cell state (32, 89). Notably, the extent of Regnase-1 edited CD19 CAR-T cell contraction appears to be model dependent, as other studies have reported sustained persistence of Regnase-1-edited TCR-transgenic and CAR-T cells following tumor clearance for many months (18, 21, 84, 90). Dual-inactivation of SOCS1 and Regnase-1 integrates the complementary enhancements of each single edit to enhance both acute anti-tumor function as well as long-term persistence and memory formation, increasing the frequency of circulating memory cells for months following tumor clearance (12, 21). Combined disruption of Regnase-1 and Roquin-1 additionally improved *in vivo* tumor accumulation and long-term persistence of human CAR-Ts (90).

Collectively, combination editing strategies, particularly those that include Regnase-1 to enhance acute T cell function, have emerged as a robust path to sustain long-term T cell function and durable anti-tumor immunity.

### 4.7 Top candidate genes and pathways

Across species, therapy formats and tumor models, CRISPR screens converge on core negative regulators of early T cell expansion, intratumoral accumulation and persistence. These include proximal and distal checkpoints of TCR signaling including the NF-kB and RNA stability pathways through targets such as DGKζ, CBLB, PTPN2, IκBα, A20, Regnase-1 and Roquin-1 as well as JAK/STAT signaling through SOCS1 and PTPN2 (**Figure 1**). Validated targets also map to GPCR signaling (Gα_s_, P2RY8), apoptosis (FAS), cell cycle/persistence (CDKN1B), and transcriptional/epigenetic fate control (IKAROS, BAF/INO80 complexes, FLI1). Experimental context matters, and effects varied by species, T cell therapy type (e.g., TCR transgenic, CAR-T, TCR-T) and tumor setting (e.g., hematological vs. solid tumor). Combinatorial editing has emerged as an effective strategy to integrate the discrete benefits of single edits and impact across the acute and persistent phases of the anti-tumor response through layered engineering, with Regnase-1 emerging as a preferred combination partner (e.g., Regnase-1/SOCS1, Regnase-1/BCOR and Regnase-1/Roquin-1). Preferred targets, whether as a single edit or in combination, should ideally remodel the Tpex/Tex balance to sustain the stem-like memory benefits of Tpex cells with the effector competence of Tex cells while diminishing differentiation into terminal exhaustion.

## 5 Design principles for CRISPR-engineered T cell therapies

Rational design should align with the patient’s tumor type, the T cell therapy format, and the full product life cycle encompassing manufacture, infusion, the acute response, and durable persistence. We propose four principles when prioritizing targets and pathways for CRISPR-engineered T cell therapies:

1. Stage-Aligned Engineering. Selected targets should match the sequential biological barriers to the T cell therapy, starting from manufacture and culminating in durable responsiveness against tumor. Edits facilitating manufacturing, expansion, post-infusion trafficking and intratumoral accumulation, resistance to tumor immunosuppression and exhaustion, and which drive durable persistence are ideally sought, with combinatorial editing useful for integrating benefits across functional phases and mitigating single-target limitations.
2. Fate Stewardship. Given the coupling of T cell differentiation fate with fulminant anti-tumor function, edits should ideally bias towards increasing Tpex and effector-competent Tex states as well as forming durable, functional memory while limiting terminal exhaustion. There should be a preference towards targets that re-shape trajectories rather than enhancing a single mechanism (e.g., just proliferation or effector function). Combinatorial editing can also be used to integrate fate benefits across disparate edits.
3. Context-Aware. Targets should be selected with consideration of T cell therapy type (CAR-T, TCR-T, TIL), targeted antigen, CAR design, and tumor context.
4. Validate Across Modalities. Given ecological limitations of *in vitro* systems, confirm hits *in vivo* with functional readout breadth (intratumoral accumulation, effector function, persistence, impact on Tpex/Tex state). Hits that reproduce across systems should be prioritized given the opportunity for cross-product synergies.

In conclusion, CRISPR screen-informed rational design of T cell therapies has the potential to overcome the intrinsic biological barrier posed by the immunosuppressive TME and help close the clinical efficacy gap in the treatment of patients with solid tumors.

## 6 Harnessing endogenous anti-tumor immunity

Tumor infiltrating lymphocytes (TIL) present within solid tumors encompass the patients’ endogenous T cell response against tumor cells. TIL are highly polyclonal and can recognize both shared and private neoantigens expressed by antigenically heterogeneous tumor cells across metastatic sites. This feature enables comprehensive tumor cell recognition by TIL and serves as the basis for TIL adoptive cellular therapies. TIL thus constitute a natural solution for solid tumors lacking broad expression of shared antigens, which constitute a vast majority of cancer patients. Tumor-reactive clonotypes present within TIL are, however, subject to the immunosuppressive mechanisms of the TME including tumor antigen-driven exhaustion, making TIL an exemplary substrate for CRISPR-informed functional engineering, where pathway edits can augment TIL function.

### 6.1 TIL therapy: clinical constraints and engineering opportunities

While TIL therapy achieves meaningful responses in unresectable, checkpoint-refractory metastatic melanoma with an approved product (Amtagvi), activity in non-melanoma indications (e.g., NSCLC, CRC, PDAC, HNSCC, cervical cancer) is more modest, highlighting tumor-context barriers, including paucity of tumor-reactive TIL and stringently immunosuppressive TMEs (91–94). The absolute dose of tumor reactive TIL, the frequency of CD8^+^ cells in the TIL product, and the product state also correlate with clinical responses (95–98). Different approaches have been explored as potential solutions to enrich tumor reactive TIL (99, 100). To further enhance TIL therapy efficacy, process levers such as shorter culture timelines, the inclusion of 4-1BB co-stimulation or use of IL-15 can shift composition and critical cellular quality attributes and create points of synergy with gene edits to steward favorable T cell fates and enhance T cell functionality in the TME (101–105). In addition to dependence on cytokines during manufacture, there is also strong mechanistic dependence of TIL therapy on JAK/STAT cytokine signaling during treatment of patients. Standard treatment regimens rely on non-myeloablative lymphodepletion (NMA-LD) prior to TIL infusion, which enhances post-infusion TIL engraftment in part by increasing the availability of cytokines such as IL-15 (106). Following infusion, patients receive high-dose IL-2 (HD-IL-2; aldesleukin), which is thought to also promote engraftment as well as initiate post-infusion expansion (107). T cells present within and reactive against tumors are antigen experienced and have committed to terminal exhaustion differentiation pathways, limiting both function as well as stemness, all while being exposed to immunosuppressive stresses. While some tumor-reactive TIL may be too far differentiated towards a terminal exhaustion fate to functionally rescue with engineering, there are tractable CRISPR screen-informed engineering opportunities to improve TIL function by targeting tumor-reactive TIL at the earliest stages of exhaustion. Engineering should ideally allow the tumor-reactive T cells present within TIL to overcome the stringent TMEs of tumor types that are poorly responsive to immunotherapies, enhancing JAK/STAT-dependent mechanisms, and rescuing stemness to improve persistence by halting and/or reversing the onset of terminal exhaustion. Critically, functional enhancements driven by engineering must manifest within the sub-population of tumor-reactive T cells present within a bulk TIL drug product.

## 7 CRISPR-enabled enhancement of TIL function

Across pooled *in vitro* and *in vivo* screens, JAK/STAT cytokine signaling, proximal/distal TCR and NF-kB signaling (including RNA-stability checkpoints), and transcriptional/epigenetic regulators of exhaustion and fate emerge as convergent levers of the mechanisms used by TIL to drive clinical benefit. Applying these insights to TIL aligns engineering with three sequential needs: i) post-infusion expansion and accumulation, ii) exhaustion resistance with preservation of Tpex-biased, effector-competent Tex subsets and iii) durable persistence and memory (**Figure 2**).

### 7.1 Modulation of cytokine signaling

Given the dependency of TIL on cytokine support during manufacture, post-infusion expansion, intra-tumoral accumulation, as well as persistence, engineering approaches enhancing JAK/STAT signaling pathways have first-order potential to improve TIL clinical activity. Indeed, SOCS1 and PTPN2 are negative regulators of JAK/STAT signaling and have repeatedly surfaced across CRISPR screens (**Figure 1**); targeting these regulators has the potential to amplify cytokine signals both during manufacture and following infusion, particularly in the TME where cytokine support is scarce. Editing SOCS1 or PTPN2 amplifies signals downstream of IL-2/IL-7/IL-12/IL-15/IL-21, serving to improve expansion and intratumoral accumulation and, in several tumor models, persistence (12, 62, 108, 109). For TIL, cytokine axis editing should raise the ceiling and durability of post-infusion expansion and intratumoral accumulation, reduce or eliminate the need for HD-IL-2 and create a permissive backdrop for fate-preservation to sustain long-term functions (**Figure 2**).

### 7.2 Fate and exhaustion control

TIL therapy is unique amongst adoptive cell therapy modalities in that the source material is comprised of antigen-experienced T cells, many of which are along the Tex differentiation fate continuum. Ex vivo manufacturing transiently reinvigorates TIL function, despite their exhausted state, suggesting that Tex is functionally modifiable, although the epigenetic scars of exhaustion are reacquired following infusion (96). In responders, induction of effector signatures not observed in non-responders underscores the importance of preventing terminal exhaustion and maintaining effector function (96). Genetic modulation of key exhaustion pathways within TIL is a potential solution to favorably modify the Tpex/Tex continuum and prevent functional exhaustion (**Figure 2**). The goal is not elimination of Tex states but stewardship of a TCF1^+^ Tpex-biased, effector-competent landscape that facilitates persistence and resists terminal exhaustion driven by tumor antigen. Combinations have the potential to optimally modulate T cell exhaustion while promoting anti-tumor effector function. Indeed, tumor-reactive TIL clones with memory-progenitor stem-like characteristics have been shown to better persist in comparison to clones with more terminally differentiated states, which declined rapidly post-infusion (110). Additionally, the phenotypic presence of CD39^-^CD69^-^ stem-like cells in infused TIL correlates with clinical responses (110). Coupling strong initial function-enhancing edits, such as achieved with Regnase-1 inactivation, together with sustained memory-enhancing edits such as SOCS1 or BCOR can integrate early expansion benefits with a long tail of maintenance stabilizing the functional reservoirs of tumor-reactive clonotypes over many months.

### 7.3 Clinical translation of target insights to TIL therapy

Applying the target insights discussed above, multiple approaches to functionally enhance TIL are now under evaluation in the clinic. For cytokine-signaling modulation, SOCS1 inactivation by gene editing is being explored in a Phase 1/2 study (KSQ-001EX; NCT06237881) (103). SOCS1/Regnase-1 dual-edited TIL are also being evaluated clinically (KSQ-004EX; NCT06598371). CISH is a negative regulator of Il-2 and IL-15 and TCR signaling, and while not occurring as a hit across CRISPR screens, inactivation in TIL has been evaluated in a Phase 1 study (NCT04426669) (111, 112). Finally, several studies are assessing PD-1–edited TIL, including IOVA-4001 (NCT06783270, NCT07035002, NCT05361174). Together, these trials will provide clinical validation of CRISPR-screen-identified targets and help complete the path from target discovery to clinical translation.

## 8 Safety considerations for enhanced gene-edited T cell therapies

Safety considerations for enhanced gene-edited T cell therapies include the impact of gene editing on genomic integrity, the nature of the engineered enhancement, and on-target/off-tumor toxicity potential.

Multiplex gene editing is achievable through high fidelity CRISPR-based editing yet brings genotoxicity risk driven by off-target edits, unintended large deletions, and chromosomal abnormalities (113, 114). Extensive off-target and genomic integrity characterization studies are required for gene edited cell products prior to and during clinical development. Recent advances in gene editing technology have improved editing precision and have eliminated a need for DNA double-stranded breaks through the development of prime and base editors as well as zinc finger repressor-driven epigenetic silencers (115, 116). Regardless of the CRISPR nuclease used, the selection of sgRNA sequences to ensure targeting specificity and desired functional disruption with minimal off-target pharmacology is critical for the development of engineered cell therapies. To facilitate sgRNA discovery, protein domain-focused CRISPR screens using sgRNA tiling libraries spanning the targeted protein have been used to accelerate sgRNA candidate discovery for therapeutic applications (12, 117).

The primary concerns with genotoxicity are unintended pharmacology driven by off-targets and the increased potential of T cells present within the infused drug product to undergo transformation. Transforming events driven by random site integrations are known to occur at extremely low frequencies with the use of viral vectors for the manufacture of approved CAR-T products (118, 119). To our knowledge, no genotoxicity-driven clinical adverse events have been observed in gene edited adoptive T cell therapies. It is also plausible that the nature of the enhancement, such the identity of the gene(s) inactivated, can also contribute to transformation risk: thus, enhancements which are also known cancer driver mutations should be altogether avoided or pursued with substantial consideration for appropriate safeguards. Lastly, the ability of an enhancement to drive uncontrolled outgrowth of cells, whether through transformation potential or due to the pharmacology of the enhancement, should be modelled and mitigated in preclinical studies.

CAR-T and TCR-T therapies can have a profound tumor-killing impact driving a strong proinflammatory milieu in the days following infusion, with cytokine release syndrome (CRS) and immune effector cell-associated neurotoxicity syndrome (ICANS) commonly associated on-target toxicities (120). By logical extension, functionally enhancing T cell therapies where these toxicities manifest may exacerbate their frequency and severity. Additionally, situations where on-target/off-tumor toxicities are clinically manageable may convert to being unacceptable. In the case of TIL therapies, nearly all clinical toxicities relate to NMA-LD prior to infusion and HD-IL-2 following, with overt CRS rare and ICANs not reported (121). Additionally, minimal intolerable on-target/off-tumor toxicities are observed, with autoimmune vitiligo and uveitis the most common manifestations in melanoma (121). This dearth of on-target/off-tumor toxicities by TIL can be in part attributed to the sustained mechanisms of central and peripheral tolerance on the bulk TIL drug product. When considering engineering enhancements for TIL therapies, those which lower the threshold for TCR activation to increase the activation status and functionality of TIL may also expand their recognized antigenic pool, facilitating broader off-tumor recognition. These safety concerns must collectively be addressed through intentional target selection and engineering approaches, pre-clinical identification of immunomodulatory agents capable of halting potential toxicities, and stepwise approaches in clinical protocol design.

## 9 Conclusions and future directions

CRISPR screens have mapped the core circuitry by which T cells achieve anti-tumor activity, repeatedly converging on JAK/STAT cytokine signaling, proximal and distal TCR/NF-κB pathways including RNA stability programs, and transcriptional/epigenetic checkpoints that shape differentiation and persistence. Looking forward, a stage-aligned, context-aware-design strategy as presented herein provides a practical approach for the selection of targets and pathways to optimally enhance T cell therapies. As dual editing of identified target combinations can integrate complementary benefits into T cell therapies, combinatorial editing will be an important tool in this approach. These insights provide a rational foundation for engineering adoptive cell therapies.

Coupling CRISPR screen-informed enhancements with the personalized and tumor-reactive features of TIL serves as a promising approach to overcome the current efficacy challenges associated with the treatment of solid tumors by T cell therapies. For TIL specifically, next-generation enhanced approaches should couple TIL-specific needs, including reducing dependence on intensive IL-2 support and attendant toxicities, with countering TME-derived suppressive cues to enhance trafficking and tumor residency. Fate-preserving edits which inhibit exhaustion and facilitate persistence ensure maximal impact on functionality of tumor-reactive T cells present within TIL.

For current knowledge gaps, available datasets likely under-sample relevant biology given a focus on CD8^+^ T cells, as CD4^+^ T cells provide help and can additionally possess direct cytotoxic capabilities. Furthermore, expanding beyond proliferation/accumulation readouts should reveal additional regulators that are difficult to capture using accumulation readouts. Translating edits from CRISPR screens into T cell therapies will require continued attention to multiplex editing and safety, and formal frameworks are still needed to interpret the frequency and clinical significance of off-target edits and chromosomal rearrangements. Beyond genome integrity, T cell fate over-steering is a concern, with edits maximizing early proliferation or effector function potentially compromising the balanced memory reservoir needed for durable responses. This underscores the value of tracking the Tpex/Tex composition and long-term persistence of engineered T cell therapies in pre-clinical and clinical studies. Finally, it will be important to define when engineering benefits plateau. Combining gene-on with gene-off approaches may enable a further leap forward in enhancement, yet optimization must extend beyond raw functionality to encompass tumor antigen recognition fidelity and clonal quality. No amount of functional enhancement compensates for insufficient tumor recognition, and enriching for TIL tumor reactivity with layered functional edits represents a clear and testable path to broaden benefit and durability across solid tumors.
